# Etched p-Type Si Nanowires for Efficient Ozone Decomposition

**DOI:** 10.1186/s11671-019-3205-6

**Published:** 2019-12-10

**Authors:** Xuan Li, Linqu Luo, Yicheng Bi, Anqi Wang, Yunfa Chen, Ning Han, Fengyun Wang

**Affiliations:** 1 College of Physics and State Key Laboratory of Bio-Fibers and Eco-Textiles, Qingdao University, Qingdao, 266071 China; 2 College of Electromechanical Engineering, Qingdao University of Science & Technology, Qingdao, 260061 China; 3 State Key Laboratory of Multiphase Complex Systems, Institute of Process Engineering, Chinese Academy of Sciences, Beijing, 100190 China

**Keywords:** Ozone decomposition, Metal-assisted chemical, etching method, Si nanowires, p-type

## Abstract

High concentration ozone can damage greatly to the respiratory, cardiovascular systems, and fertility of people, and catalytic decomposition is an important strategy to reduce its harm. However, it remains a challenge to develop efficient ozone decomposition catalysts with high efficiency. In this study, p- and n-type silicon nanowires (Si NWs) are fabricated by wet chemical etching method and are firstly applied to catalytic decompose ozone at room temperature. The p-type Si NWs exhibit 90% ozone (20 ppm O_3_/air) decomposition efficiency with great stability, which is much better than that of n-type Si NWs (50%) with same crystal orientation, similar diameter and specific surface area. The catalytic property difference is mainly attributed to the more delocalization holes in the p-type Si NWs, which can accelerate the desorption of ozone decomposition intermediates (i.e., adsorbed oxygen species).

## Highlights

- High quality Si NWs were prepared by a rapid, facile, and lost cost MACE method.
- Si NWs were first applied to catalyze ozone decomposition.
- P-type Si NWs possess better ozone decomposition performance than that of n-type Si NWs, and the relative catalytic mechanism is explained.

## Introduction

Due to the strong oxidative properties, ozone can react with most of the proteins and nucleic acids and thus is widely used in sterilization, pulp processing, and decomposing pollutants [1]. However, the strong oxidative properties of ozone induce a lot of adverse effects on the human body such as damaging respiratory tract, cardiovascular and fertility [2–5]. At present, indoor ozone which usually is generated by ultraviolet irradiation is still one of the most prominent air pollutants all over the world. In order to reduce indoor ozone concentration, a variety of decomposition catalyst have been synthesized, including activated carbon-based materials [6], noble metal materials [7–9], and transition metal oxides [10–12]. However, the relationship between catalyst property and degradation performance is not well discovered, and the highly active catalyst preparation is still challenging.

As one-dimensional semiconductors, silicon nanowires (Si NWs) with high specific area, excellent physical, and chemical stability, are widely applied in solar cells, lithium-ion batteries, photocatalysts, and so on [13, 14]. In this work, both p- and n-type Si NWs are prepared by a rapid and facile metal–assisted chemical–etching (MACE) method [15], and are applied to catalytic decomposition of ozone. The results show that the p-type Si NWs exhibit high decomposition efficiency (> 90%) with great stability in a 16 h test to 20 ppm ozone, which is much better than that of n-type Si NWs (~ 50% after 12 h). This work exhibits the advantage of p-type Si in ozone decomposition behaving as an electron trap to facilitate the intermediate O_2_^2−^ desorption, and also a new application of p-type Si NWs for highly active ozone decomposition catalyst.

## Materials and Methods

### Fabrications of Si NWs

The p- and n-type Si (100) wafers with the resistivity of 1–10 Ω cm were cut into 2 × 2 cm^2^ squares, washed with deionized (DI) water, ethanol, and acetone under sonication in turn for 15 min. Then the cleaned Si wafers were immersed in a mixed solution containing H_2_SO_4_ (97%) and H_2_O_2_ (35%) in a volume ratio of 3:1 for half an hour to remove organic impurities. After that, the Si wafers were immersed into the 5% HF solution for 3 min to form Si–H bonds. Then the wafers were immediately placed into a solution of 0.005 M AgNO_3_ and 4.8 M HF for 1 min to coat Ag nanoparticles acting as etching catalysts. In order to guarantee the quality of the Si NWs, wafers were washed with DI water to remove the redundant Ag^+^ and then transferred into a solution containing 4.8 M HF and 0.4 M H_2_O_2_ in dark and room temperature for 1 h in order to get enough length of the NWs.

### Catalyst Characterization Catalytic Activity Tests

The morphology of the sample with various type Si NWs was characterized by scanning electron microscopy (SEM, JEOL JSM-7800F, Tokyo, Japan). Furthermore, the crystal microstructures of p (100) and n (100) Si NWs were studied with transmission electron microscopy (TEM, Philips Technai 12 under 80 kV operation voltage, Amsterdam, Netherlands) and high resolution TEM (HRTEM, Philips CM200 under 200 kV operation voltage, Amsterdam, Netherlands). For ozone catalytic performance testing, the Si NWs were scraped from the intact wafers by a razor blade, and 50 mg Si NWs were mixed with 450 mg quartz sand. The mixture was then placed in a U-shaped tube reactor, introducing with 20 ppm ozone generated by COM-AD-01-OEM generator (Anshan, China). In the absence of humidity, the total space velocity (SV) was 240,000 mL g^−1^ h^−1^ with a gas flow of 200 mL min^−1^, and the concentration was detected by 106 M monitor (2B technology, USA).

## Results and Discussion

**Fig. 1:**
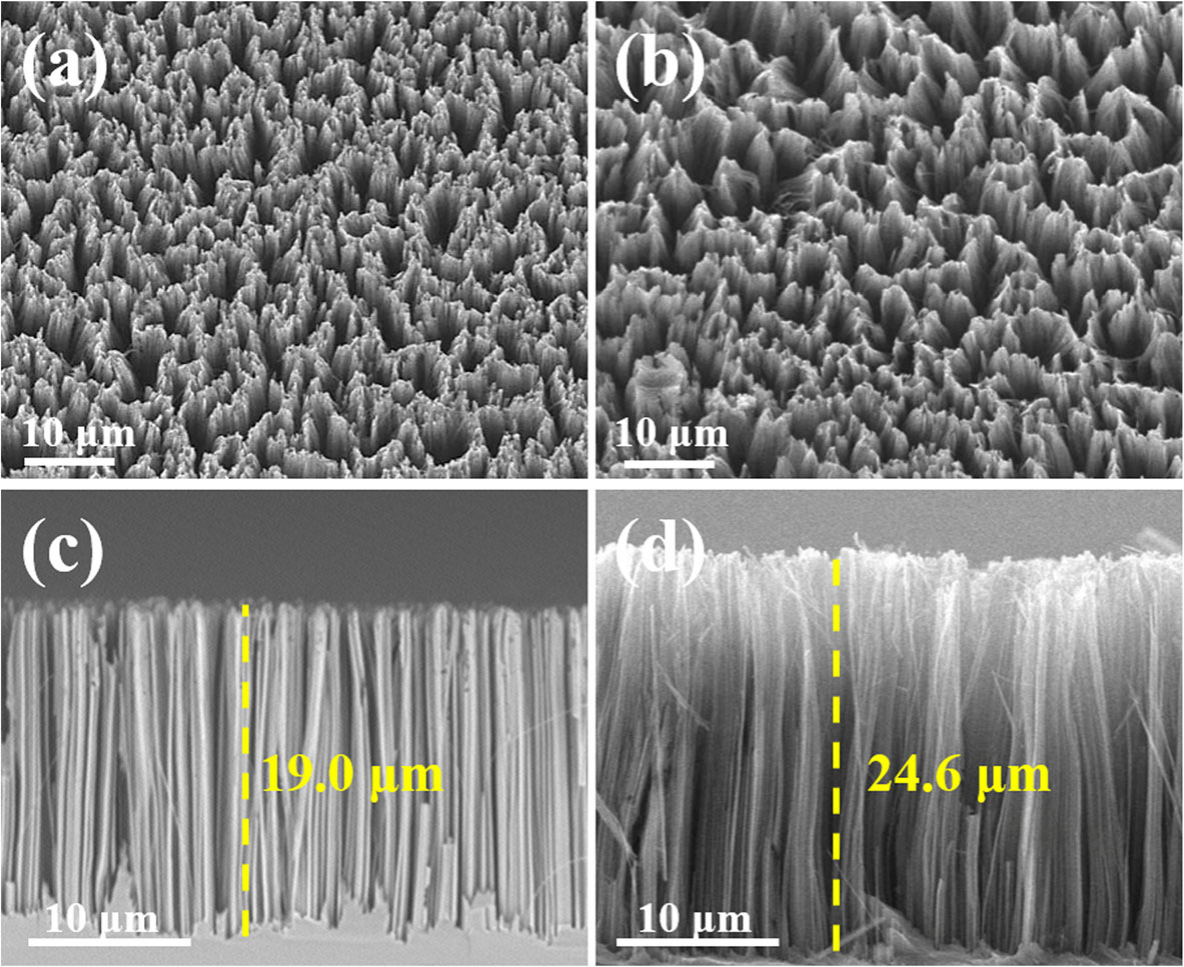
SEM images of the catalysts: **a** and **c** the top view and sectional view of p-type Si NWs; **b** and **d** the top view and sectional view of n-type Si NWs

In order to fully understand the surface morphology of Si NWs, SEM was applied to characterize the obtained samples (Fig. 1). The top-view SEM image of the p (100) Si NWs arrays is shown in Fig. 1a, which demonstrates that Si NWs are uniformly distributed on the surface of Si wafers. Figure 1c shows the cross-section drawn of p (100) Si NWs arrays, demonstrating that the Si NWs on the surface of the Si substrate are uniform. It is noted that the n (100) NWs (about 24.6 μm) are a bit longer than p (100) NWs (about 19.0 μm), which is resulted by the relatively faster etching rate. In the oxidation etching process, the silicon wafers are firstly oxidized into silicon oxide and then the oxidized substance lost some electrons after being etched by HF. N-type silicon wafers have much more electrons than p-type silicon wafers. Therefore, the oxidation rate of n silicon wafers is greater than that of the p-type silicon wafers, which will make the etching reaction rate faster for n-type and thus the n-type silicon nanowires are longer than that of p-type silicon nanowires within the same etching time. The top-view and cross-section SEM images of n (100) Si NWs arrays are showed in Fig. 1b and d. The top-view of n (100) Si NWs is similar to that of p (100) Si NWs. The two kinds of NWs are both highly uniform and dense, with similar density of ~ 10^10^ cm^−2^.

**Fig. 2:**
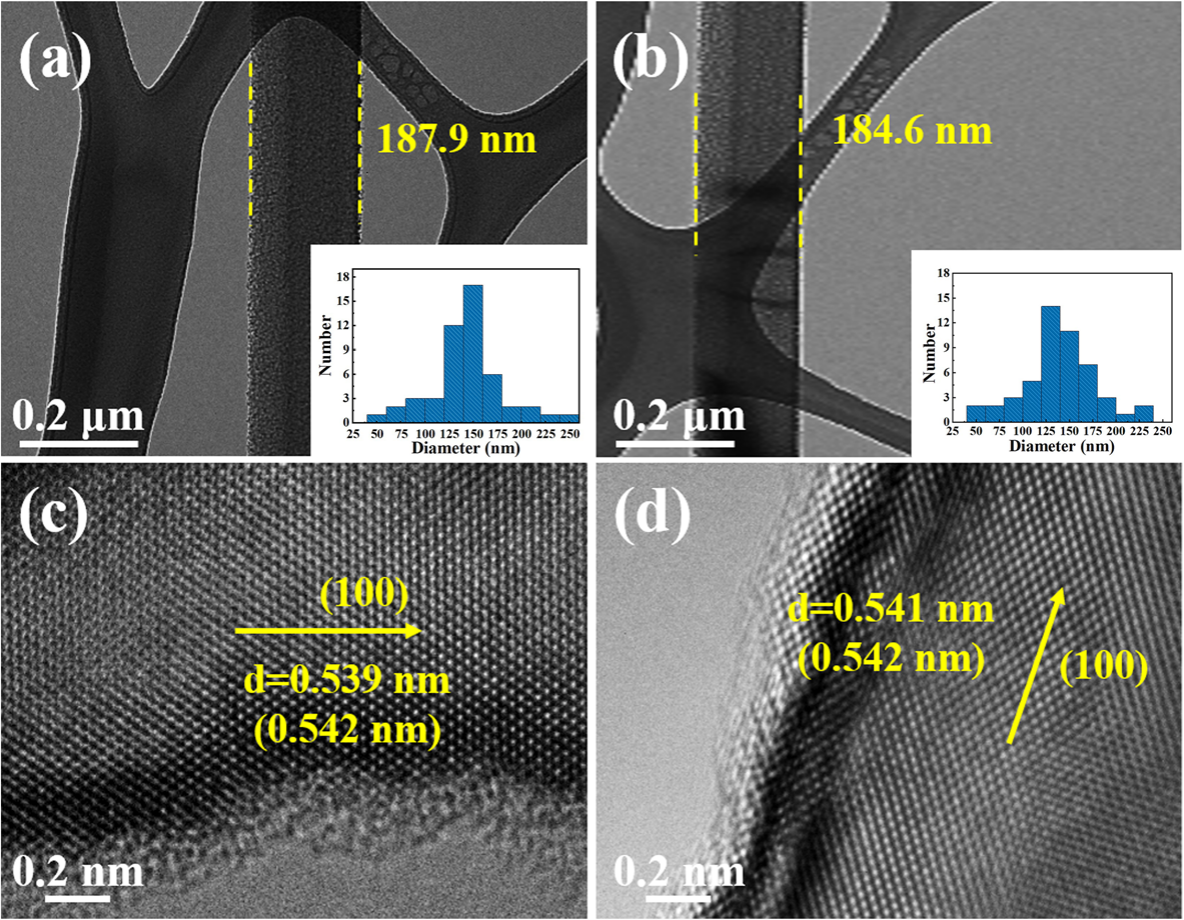
TEM and HRTEM images of catalysts: **a** and **c** p-type Si NWs **b** and **d** n-type Si NWs illustrations are the normal distributions of diameter corresponding to the p (100) NWs and n (100) NWs, respectively

For further understanding of the specific morphology of single Si NW, the TEM images of single NW for p (100) and n (100) are shown in Fig. 2. Obviously, both p-type and n-type Si NWs have relative smooth surface, with the diameter of 187.9 nm and 184.6 nm, respectively. In order to get the diameter distribution of Si NWs, the statistical results based on the diameters of 50 Si NWs are shown in the illustrations of Fig. 2a and b, respectively. The histograms show that the diameters of p and n (100) NWs mainly fall in the range of 125~175 nm, which ensures the similar specific surface areas (12.68 m^2^/g and 13.66 m^2^/g), and further ensure a consistent comparison of the catalytic performance in this study. The corresponding HRTEM images (Fig. 2b and d) show the interplanar spacings of p- and n-type Si NWs is 0.539 nm and 0.541 nm, respectively, very close to the theoretical value of 0.542 nm ((100) lattice plane). It is revealed that both p- and n-type Si NWs extend along the <100> direction, which is consistent with the etched Si substrate. The HRTEM image also shows that there are no obvious holes and defects in the Si NWs.

**Fig. 3:**
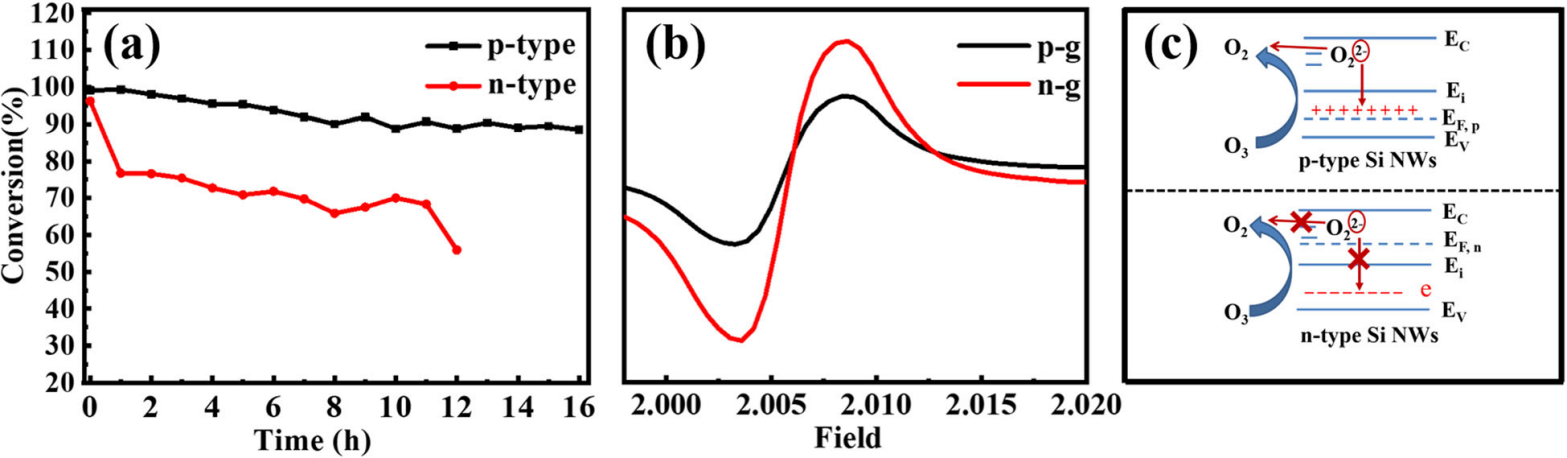
**a** The conversion of ozone using p- and n-type Si NWs as catalysts; **b** EPR detection of p- and n-type Si NWs; and **c** Schematic comparison of p- and n-type Si NWs. P-type Si is lack of electrons and has positively charged holes, and thus, the release of electron and desorption of O_2_^2−^ is easier than the case of n-type Si

The results of the catalytic activity of Si NWs are shown in Fig. 3, which were tested by decomposing 20 ppm ozone in air carrier at room temperature. Compared with n-type Si NWs, p-type Si NWs show better catalytic ozone decomposition performance with high initial efficiency of ~ 99%, and then the efficiency decreases slightly over time and remains > 90% after 16 h. As for the catalytic activity of n (100) Si NWs, its initial efficiency is approximately 96%; however, the efficiency decreases relatively rapidly, and it only remains ~ 50% after 12 h testing. The outstanding catalytic performance of p (100) Si NWs is consisted with the regularity that the semiconductors with strong p-type behavior present high performance for ozone decomposition [16]. Notably, the p- and n-type Si NWs were prepared under exactly the same condition. Also both the two types Si NWs have the same growth direction, similar specific surface areas and diameter distribution. All of these indicate that the difference between two types Si NWs comes only from their semiconductor type.

According to the mechanism proposed by Oyama et al. [12] the ozone catalytic decomposition process can be divided into the following steps:

1 $$ {\mathrm{O}}_3+\ast \to {\mathrm{O}}_2+{\mathrm{O}}^{\ast } $$

2 $$ {\mathrm{O}}_3+{\mathrm{O}}^{\ast}\to {{\mathrm{O}}_2}^{\ast }+{\mathrm{O}}_2 $$

3 $$ {{\mathrm{O}}_2}^{\ast}\to {\mathrm{O}}_2+\ast \left(\mathrm{slow}\right) $$

Here, the symbol * represents an active site, and the release rate of active oxygen in (3) from catalyst determines the overall rate of ozone decomposition. In our previous work, the intermediate products have been confirmed to peroxide species (O_2_^2−^) determined by Raman spectroscopy [10]. The main reason for the high catalytic efficiency and excellent stability of p-type Si NWs is that they have more delocalized positively charged holes, which are beneficial to attracting electrons from adsorbed O_2_^2−^ anions. Then, the intermediate oxygen species are easy to desorb from the catalyst, exposing the active site again for continuous ozone decomposition [17]. This is further proved by the electron spin resonance (EPR) measurement as shown in Fig. 3b. A sharper signal at g = 2.0052 is detected in n-type Si NWs than that of p-type ones, indicating that the n-type Si NWs have more suspended bonds [18], which makes them have a stronger adsorption interaction with oxygen molecules. As a result, the adsorbed oxygen molecules are not desorbed quickly, which occupy the active sites resulting in the n-type Si NWs more vulnerable to be deactivated, as schematically shown in Fig. 3c.

## Conclusion

In summary, the p-type Si NWs prepared by the MACE method exhibit high ozone conversion efficiency of > 90% after 16 h test at room temperature. The outstanding catalytic performance can be mainly attributed to a mass of delocalized holes, which is beneficial to the electron release of ozone degradation intermediate (O_2_^2−^) and thus favor its desorption during ozone decomposition. All these have indicated the great potency of the p-type Si NWs for ozone-catalyzed decomposition, especially after further optimization in the future.

## Data Availability

The datasets supporting the conclusions of this manuscript are included within the manuscript.

## Abbreviations

DI: Deionized
EPR: Electron spin resonance
HRTEM: High resolution transmission electron microscopy
MACE: Metal-assisted chemical etching
SEM: Scanning electron microscopy
Si NWs: Silicon nanowires
TEM: Transmission electron microscopy
